# Understanding the local environment in electrocatalysis

**DOI:** 10.1093/nsr/nwae250

**Published:** 2024-07-26

**Authors:** Chaojie Chen, Yao Zheng, Shi-Zhang Qiao

**Affiliations:** School of Chemical Engineering, The University of Adelaide, Australia; School of Chemical Engineering, The University of Adelaide, Australia; School of Chemical Engineering, The University of Adelaide, Australia

## Abstract

This perspective summarizes the understanding about the local reaction environment in the electrocatalysis and underscores the influence of local environment due to its special location.

The core of electrolysis is utilizing electrocatalysts to reduce the activation energy and enhance the rate of electrocatalytic reactions. Nowadays, quantitative efforts are being conducted towards modifying catalysts to tune the electronic structure because an ideal electronic structure can change the reaction pathways and improve the conversion rate. Besides, the electrode–electrolyte interface (EEI), where electron and charge transfer processes occur, is another key factor affecting the surface electrochemical reaction's rate [1]. Delving into the interface is imperative for deepening our understanding and expediting the industrial integration of electrocatalytic reactions. The nanoscale space at/near the EEI is known as the local environment, also known as the microenvironment [2]. The local environment directly interfaces with electrocatalysts. Therefore, the kinetic process at the interface is greatly determined by the electronic structure of the electrocatalyst, while the diffusion and transport processes in the local environment are primarily affected by the electrolyte. With changes in electrocatalyst's electronic structure and electrolyte systems, the inner of the local environment undergoes intricate dynamic evolution. Its structure can be subdivided into Stern layer (it is composed of the inner Helmholtz plane (IHP) and outer Helmholtz plane (OHP)) and Diffuse layer (Fig. 1). The IHP is composed of ions specifically adsorbed on the electrode surface, mainly solvent molecules. In the OHP, the solvated ions from the electrolyte are distributed in this region to approach the electrode. Therefore, the surface states, such as interfacial field distribution, significantly affect the IHP, while the OHP is more sensitive to the change in solvent properties. During the electrocatalytic reaction process, intermediates primarily undergo evolution and migration processes within the Stern layer. Simultaneously, reactants and products diffuse through the diffuse layer to access the catalyst surface and bulk electrolyte. Therefore, the structure of the local environment significantly influences the internal reaction and transfer process. Studying the molecular-scale structure of the local environment and its dynamic response to external conditions is crucial for achieving efficient matching between the electrocatalyst and the electrolyte.

**Figure 1.**
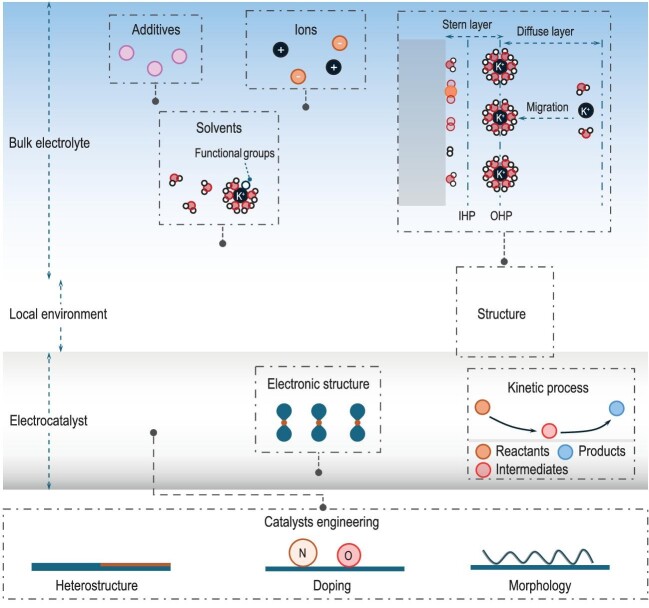
The electrochemical system can be divided into three parts: electrocatalysts, local environment, and bulk electrolyte. The electronic structure of electrocatalysts affect the kinetics process at the electrolyte-electrode interface, while the electrolyte system influences the diffusion rate of species. Meanwhile, the changes in electrocatalysts and electrolyte will result in the dynamic evolution of the local environment. The structure of the local environment is composed of a Stern layer and Diffuse layer. The Stern layer can be subdivided into IHP (inner Helmholtz plane) and OHP (outer Helmholtz plane). The reactants will interface with electrodes and evolve in this region.

The influence of electrocatalysts on the local environment primarily manifests in their electronic structure. Electronic structure refers to the distribution of electron energy levels, and it is related to the binding energy of adsorbed species, which directly determines overall activity and selectivity (Fig. 1) [3]. The catalytic activity exhibits a volcano relationship with the binding energy of adsorbed species, where moderate binding energy can achieve the highest catalytic performance [4]. Therefore, the challenge in catalysis research lies in precisely controlling the electronic effects to achieve the most suitable adsorption strength for key intermediates. Since Markovic and Nørskov proposed the concept of the ‘metal d-band center’, altering the interfacial structure to modify electronic structure has become a focal point in electrocatalyst engineering. Nowadays, various strategies, such as doping, defect/morphology engineering, valence state regulation, etc., have been employed to modulate surface structure for improving catalytic activity [5,6]. These modification methods offer effective ways to adjust the electronic structure, consequently leading to changes in the Fermi-level of catalysts. Such alterations in the Fermi level exert influence over the local electric field, thereby driving the dynamics evolution of the local environment and affecting adsorption of the intermediates.

The electrolyte plays a crucial role in the local environment through its impact on the electric double layer (EDL) structure. The compositions in the local environment are derived from the electrolyte solution, while its structure differs significantly from that of bulk solution (Fig. 1). For example, in reactions involving the consumption of protons/hydroxide ions, the local environment adjacent to the electrode accumulates hydroxide ions/protons, leading to the formation of a local pH in the local environment. General strategies for modifying electrolyte include solvents, ions (cations and anions) and additives. Solvents act as carriers and determine the solubility and ionic conductivity of electrolyte salts. The aprotic solvents feature for various functional groups and physicochemical properties. The introduction of aprotic solvents can effectively confine water into strong hydrogen bond networks [7]. The anions and cations are essential parts in the electrolyte. The absence of cations in the solution will lead to a rapid decline in the electric conductivity [8]. The introduction of cations can enhance the local electric field in the local environment, which hinders the transport of hydroxonium ions and improves local pH. Moreover, it is generally recognized that the presence of solvated cations is beneficial for the stabilization and activation of key intermediates. The anions (e.g. halides) can buffer the solution or induce the reconstruction of the surface. The additive strategy is also an effective way to modify the electrolyte solution. The inner mechanism is proposed as abundant functional groups in the additive that can enhance/weaken interactions between key intermediates and the interface [9]. Further studies suggest that additives affected the solvated structures of cations, thereby enhancing the electric field at the EEI.

The special position of the local environment underscores the profound influence of both catalyst's electronic structure and electrolyte composition. However, two key problems need be addressed for further developments in this field: (i) deepening our understanding of how electronic structure can impact the local environment; (ii) studying the mechanism about the influence of electrolyte on the dynamic's evolution in the local environment. We hold a belief that deep understanding about the local environment will be beneficial for the enhancement of electrocatalyst and electrolyser's durability. Creating a moderate local environment will be of great significance for the commercial development of electrocatalysis.
